# CRISPR-Cas9 cleavage efficiency correlates strongly with target-sgRNA folding stability: from physical mechanism to off-target assessment

**DOI:** 10.1038/s41598-017-00180-1

**Published:** 2017-03-10

**Authors:** Xiaojun Xu, Dongsheng Duan, Shi-Jie Chen

**Affiliations:** 1 Department of Physics, Department of Biochemistry, and Informatics Institute, University of Missouri, Columbia, MO USA; 2 Department of Molecular Microbiology and Immunology, Department of Neurology, School of Medicine; Department of Biomedical Sciences, College of Veterinary Medicine; and Department of Bioengineering, University of Missouri, Columbia, MO USA

## Abstract

The CRISPR/Cas9 complex, a bacterial immune response system, has been widely adopted for RNA-guided genome editing and transcription regulation in applications such as targeted genome modification and site-directed mutagenesis. However, the physical basis for its target specificity is not fully understood. In this study, based on a statistical mechanical analysis for the whole ensemble of sgRNA-target complex conformations, we identify a strong correlation between Cas9 cleavage efficiency and the stability of the DNA-RNA (R-loop) complex structures, with a Pearson correlation coefficient ranging from 0.775 to 0.886 for the tested systems. The finding leads to quantitative insights into important experimental results, such as the greater Cas9 tolerance to single-base mismatches in PAM-distal region than to PAM-proximal region and the high specificity and efficiency for shorter protospacers. Moreover, the results from the genome-wide off-target assessments, compared with other off-target scoring tools, indicate that the statistical mechanics-based approach provides more reliable off-target analyses and sgRNA design. To facilitate the genome engineering applications, a new web-based tool for genome-wide off-target assessment is established.

## Introduction

The clustered regularly interspaced short palindromic repeats (CRISPR)/Cas9 system^1–9^, as a simple but efficient genome editing tool, has attracted increasing attentions recently. The Cas9/sgRNA (single guide RNA) has recently been used for effective gene targeting in many organisms/cells. For example, CRISPR/Cas9 *in vivo* gene editing leads to improved muscle function in a mouse model of Duchenne muscular dystrophy (DMD)^10^. Such results have established CRISPR/Cas9-based genome editing as an effective tool for gene modification in skeletal and cardiac muscle, and as a therapeutic approach to treat neuromuscular disorders and potentially many other diseases. However, considerable off-target effects hinder the application of this technology and inspire development/improvement of this technology to enhance the safety and efficacy of the promised genetic disease treatment.

**Figure 1:**
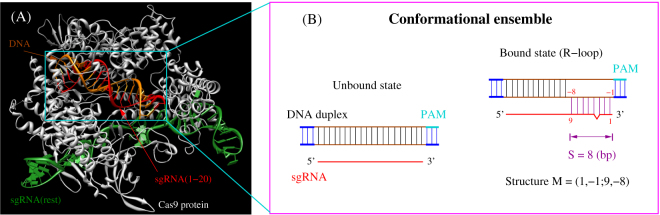
(**A**) The crystal structure of SpCas9 in complex with guide RNA and target DNA^15, 16^. The sgRNA is tightly bound with the Cas9 protein. The first 20-nt of sgRNA (red) is base paired with the DNA target (orange) and the rest sgRNA nucleotides (green) have rich interactions with the Cas9 protein. As a result, we can only consider the DNA/RNA system (in the cyan box) to model the process of sgRNA binding to the DNA target site. (**B**) The competition between base stacking within the DNA and DNA-RNA hybrid base pairing/stacking results in the different DNA/RNA bound structures. In the unbound state, the target site maintains its original DNA-DNA base pairing within the chromatin. In the bound state, the sgRNA invades into the DNA duplex and forms the R-loop structure with the target DNA. The three-base pair DNA helix stretches on both ends of the R-loop are shown in blue. We allow a single-base bulge to be formed in the hybrid duplex. *S* denotes the length (number of base pairs) of the hybrid helix.

Recent experimental and theoretical studies^11–13^ proposed a two-state model for Cas9/sgRNA binding and cleavage: PAM recognition and R-loop formation (see Fig. 1). PAM recognition is governed by the PAM sequence such as NGG for the Streptococcus pyogenes Cas9 (SpCas9) and the PAM-proximal sequences. The improved specificity^14^ with an engineered Cas9 nucleases supports the importance of PAM recognition to the overall Cas9/sgRNA mechanism. Here, the design of the Cas9 nucleases is based on the crystal structure of spCas9 in complex with guide RNA and target DNA^15, 16^ and uses the strategy of charge neutralization in the PAM-interacting non-target strand groove.

The physical mechanism for the CRISPR activity is not fully understood and the accuracy for quantitative predictions for any given Cas9/target/sgRNA system is not always reliable. The targeting specificity of Cas9/sgRNA is regulated by the types of the PAM (protospacer adjacent motif) and the nature of mismatches in different regions of the protospacer in the target site. Many currently available tools are based on the mismatch information, such as the number and the position of mismatches, to find and evaluate potential off-target sites. For example, CROP-it^17^ scores the potential off-target sites by dividing the protospacer into three segments with weight coefficients trained/optimized with the experimental (ChIP-Seq) data. CCTop^18^ and Zhang’s model (http://crispr.mit.edu/)^19^ employ position-dependent weight coefficients in their off-target scoring algorithms. Recently, Doench *et al*.^20, 21^ developed a mismatch sequence- and position-dependent off-target scoring tool, namely, the Cutting Frequency Determination (CFD) score, with 240 fitting parameters. The recent findings of the off-target activity for sequences with more general insertions and deletions between target DNA and guide RNA^22^ suggest the need for a new tool that can treat sequences beyond simple mismatches. Here we report such a new tool. This new computational tool is developed based on rigorous physical principles, can reproduce the previous experimental data, and can offer a general and consistent method for genome-wide off-target assessment and rational design of sgRNAs.

Quantitative predictions of the CRISPR activity require understanding of the physical mechanism. We reason that CRISPR-guided cleavage depends on the formation of active sgRNA-DNA state and such an active state may form an ensemble of structures. One of the distinctive features of our new method is the consideration of an ensemble (instead of a single structure as did in previous approaches) of active structures of the sgRNA-DNA complex. In our approach, we first identify the active sgRNA-DNA structures using experimentally determined CRISPR activity data. Based on the identified active structures, we establish a quantitative relationship between structure, stability, and the CRISPR activity using statistical mechanical analysis. The analysis further leads to a new predictive tool with two significant applications: (a) for a given target, to predict CRISPR activities for the different sgRNA sequences, and (b) for a given sgRNA, to provide genome-wide prediction for the potential off-target sites.

## Methods

### Test cases with single mutations

Recent experiments^14, 19, 23, 24^ showed that the CRISPR/Cas9 system can tolerate sgRNA-DNA mismatches and the gene editing efficiency is sensitive to the number, position and distribution of the mismatches. Zhang and his colleagues^14, 19^ chose four target sites within the human EMX1 gene (1, 2, 3, and 6) and one target site within the VEGFA(1) gene, and for each, generated a set of 57 different guide RNAs, which contains all the possible single-nucleotide substitutions in positions 1–19 directly 5′ of the requisite NGG PAM (see SI for an example). The 5′ guanine at position 20 is preserved, since the U6 promoter requires guanine as the first nucleotide of its transcript. These “off-target” guide RNAs were then assessed for cleavage activity at the on-target genomic locus. Qi *et al*.^23^ and Liu *et al*.^24^ studied mRFP(NT1) in E.coli MG1655 genome and the Renilla luciferase gene, respectively, with single-nucleotide substitutions (A ↔ U, G ↔ C) in positions 1–20 directly 5′ of the requisite NGG PAM. The data from these target sites provide ideal test cases for the investigation of sgRNA-DNA binding after PAM recognition. For this reason, we opt to use these published data in our analysis. Specifically, the data includes the target site DNA sequence, the perfectly matched sgRNA (without mismatch) and 57/20 sgRNAs that contain single mutation, and Cas9 cleavage efficiency from all seven target sites^14, 19, 23, 24^. The protospacers in all the targets are 20-nt in length.

### DNA-sgRNA structural ensemble

From the crystal structure of SpCas9 in complex with the guide RNA and the target DNA^15, 16^ (see Fig. 1A), we find that the sgRNA is tightly bound with the Cas9 protein, where the 20-nt single-stranded guide sequence is wrapped around by the Cas9. To access the target, the guide sequence of sgRNA is likely in the single-stranded state before binding, which suggests that we can ignore the formation of the self-structure of the sgRNA in the sgRNA-DNA complex. Furthermore, the strong stability of DNA helix suggests that we can ignore DNA structural changes outside the R-loop region.

After PAM recognition (cyan in Fig. 1B), the sgRNA randomly searches for the binding sites on the target DNA strand for RNA-DNA hybridization, resulting in different sgRNA-DNA complex structures. A sgRNA-DNA bound state corresponds to all the structures where the sgRNA and the DNA are bounded by at least one base stack (minimum hybrid helix). The bound state includes partially as well as fully zipped (20-bp) DNA-RNA duplexes. Furthermore, as shown in Fig. 1B, we define a DNA-sgRNA binding mode “M” by the terminal base pairs of the duplex (See Fig. 1B). For example, M = (1, −1; 9, −8) in Fig. 1B denotes the duplex closed by terminal base pairs (1, −1) and (9, −8). We allow the formation of a single-nucleotide bulge^22^ and the bulged nucleotide can be either on the RNA strand or on the DNA strand.

The sgRNA-DNA binding process involves competition between DNA-DNA and sgRNA-DNA base pairing. Complete DNA-DNA pairing results in the unbound state of the sgRNA-DNA system (Fig. 1B). The pairing of sgRNA with DNA causes the R-loop formation and changes the system from the unbound state to the bound state. The probability for sgRNA-DNA hybridization (binding) is determined by the free energy difference between the bound and the unbound states.

#### Free energy of the unbound state

In the unbound state, the free energy of the (separated) sgRNA and DNA is the sum of the (unbound) DNA and sgRNA: Δ*G*_*unbound*_ = Δ*G* ^(*DNA*)^ + Δ*G* ^(*RNA*)^. The DNA duplex free energy Δ*G* ^(*DNA*)^ is the sum of the experimentally determined base pairing/stacking free energy parameters^25^. The free energy Δ*G* ^(*RNA*)^ of the sgRNA, which is assumed to be a single-stranded random coil in the unbound state, is set to be zero as the reference state.

#### Free energy of the bound state

The DNA-sgRNA binding involves two steps: disruption of the DNA duplex and the subsequent DNA- sgRNA base pairing (R-loop formation). The free energy for a given bound state “M” is the sum of the free energy changes in the two steps: ${\rm{\Delta }}{G}_{M}={\rm{\Delta }}{G}_{M}^{(DNA)}+{\rm{\Delta }}{G}_{M}^{(hybd)}$, where ${\rm{\Delta }}{G}_{M}^{(DNA)}$ and ${\rm{\Delta }}{G}_{M}^{(hybd)}$ are the free energy changes in the DNA duplex and the sgRNA-DNA duplex, respectively. The sum over all the possible bound states gives the total bound free energy: ${\rm{\Delta }}{G}_{bound}=\,{\sum }_{M}{e}^{-{\rm{\Delta }}{G}_{M}/{k}_{B}T}$.

#### Folding stability

For the system shown in Fig. 1, there exist one unbound structure and 17,974 bound structures^26, 27^. The sum of all the bound and the unbound states gives the total free energy Δ*G*_*tot*_ of the sgRNA/DNA system: ${\rm{\Delta }}{G}_{tot}=-{k}_{B}Tln({e}^{-{\rm{\Delta }}{G}_{unbound}/{k}_{B}T}+{e}^{-{\rm{\Delta }}{G}_{bound}/{k}_{B}T})$. The folding stability of the bound state “M” can be characterized by the free energy difference between the bound state “M” and the total state: ${\rm{\Delta }}{G}_{M}^{f}={\rm{\Delta }}{G}_{M}-{\rm{\Delta }}{G}_{tot}$, or, equivalently, the fractional population, which is the exponential of the free energy difference: ${P}_{M}={e}^{-{\rm{\Delta }}{G}_{M}^{f}/{k}_{B}T}$.

#### Search for functional structures

Not all the bound structures lead to successful Cas9 cleavage. To search for the active bound structures, we compute the total population of all the putative active structures:

1 $${P}_{{\rm{active}}}={\sum }_{M,{\rm{active}}}{P}_{M}$$

and test the correlation between the population *P*_*active*_ and the cleavage efficiency *F* using the Pearson correlation coefficient r(*P*_*active*_, *F*). Here the Pearson correlation coefficient between parameters *x* and *y* is defined as

2 $$r(x,y)=\frac{{\sum }_{i=1}^{n}({x}_{i}-\bar{x})({y}_{i}-\bar{y})}{\sqrt{{\sum }_{i=1}^{n}{({x}_{i}-\bar{x})}^{2}}\sqrt{{\sum }_{i=1}^{n}{({y}_{i}-\bar{y})}^{2}}}$$

where $\bar{x}={\sum }_{i=1}^{n}{x}_{i}/n$ and $\bar{y}={\sum }_{i=1}^{n}{y}_{i}/n$ (the sample mean), *n* is the number of test systems (DNA/RNA sequences), *x*_*i*_ and *y*_*i*_ are the parameter values in the *i*-th test set.

A complication of the above analysis arises from the fact that not all the mismatch free energy parameters for the RNA-DNA hybrid helix are available^28^. To minimize the uncertainty in mismatches parameters in our search for the active structures, we use the forementioned published data sets^14, 19, 23, 24^, which involve at most one mismatch in each case, to search for the active structures. For these prototype systems, as an approximation, we simply assign zero free energy to a mismatched stack, which is less stable than a canonical base stack (usually with a negative free energy). Because the systems contain only a single mutation/mismatch, the free energy of the structures is mainly determined by the majority canonical base pairs/stacks rather than the single mismatch (see SI for details).

#### Assessment of off-target sites

To predict off-target sites for a given sgRNA, we need to account for the sequence-dependent energetic contributions from different mismatches, including tandem mismatches. To better assess genome-wide off-targets, we introduce nucleotide type and position-dependent mismatch parameters for DNA-RNA hybrid base pairs/stacks and one-bulge loop parameter in the DNA-RNA hybrid helix (see SI for details). We use the experimentally determined genome-wide off-target activity data^11, 19, 29, 30^ and a random search algorithm^31, 32^ to optimize the parameters. Specifically, for a given sgRNA, we sample all the possible targets in the genome and rank them according to the binding affinity of the functional sgRNA-DNA structures. By maximizing the Pearson correlation coefficient between the theoretically predicted binding affinity and the experimentally measured Cas9 cleavage efficiency on the different targets, we extract a set of mismatch parameters. As shown in the results section and the SI, the extracted parameters lead to great improvements in the predictions of Cas9 cleavage efficiency.

## Results and Discussion

### Putative sgRNA/DNA/Cas9 functional structures

We test three types of putative functional structures. The first candidate is the bound structure with the fully zipped 20 base pairs between the sgRNA and the DNA target, i.e., the bound structure (1, −1; 20, −20) in Fig. 1. We denote this candidate as the “full-zip” structure. The second candidate is the ensemble of all the bound structures, denoted as the “bound” structure. The third candidate is the hybrid helix starting immediately upstream of the PAM sequence containing the (1, −1) base pair and a helix no shorter than the minimum length of *S*_*min*_ base pairs. We denote this candidate as the “active” structures. As shown below, we find that the “active” structure (candidate 3 above) with *S*_*min*_ = 7 may be the functional structure.

### Structure-activity correlation

We identify the functional structure as the one that yields the maximum structure-activity correlation r(*P*_*active*_, *F*). As shown in Table 1, there exist no consistent correlation between the cleavage efficiency and the free energy of the fully zipped 20-bp structure (candidate 1 above). The finding is consistent with the result in the previous study^13^. In the tests for candidate 2 above (ensemble of all the bound structures), we find a correlation of 0.782 for the target of EMX1.1 and −0.426 for the target of VEGFA.1. The inconsistent results suggest that after PAM recognition, not all the bound structures can lead to efficient cleavage by the Cas9 protein.

**Table 1:** Comparison between the current new method and four other existing methods; CROP-it^17^, CCTop^18^, Zhang’s model^19^, and CFD^21^.

Target	Δ*G* ^*helix*^	*P*_*full*–*zip*_	*P*_*active*_	*P*_*bound*_	CROP-it	CCTop	Zhang’s model	CFD
EMX1.1	−0.208	−0.017	**0**.**798**	0.782	0.713	−0.693	0.74	0.719
EMX1.2	−0.072	0.029	**0**.**789**	0.532	0.658	−0.72	0.755	0.799
EMX1.3	0.188	0.015	**0**.**802**	0.387	0.72	−0.714	0.727	0.463
EMX1.6	0.503	−0.007	**0**.**831**	−0.046	0.755	−0.58	0.728	0.678
VEGFA.1	−0.074	0.148	**0**.**886**	−0.426	0.799	−0.637	0.724	0.736
Renilla	−0.132	0.359	**0**.**856**	0.201	0.781	−0.864	0.603	0.355
mRFP	−0.554	0.512	**0**.**775**	0.624	0.769	−0.759	0.451	0.674
Average	−0.050	0.148	**0**.**82**	0.293	0.742	−0.71	0.675	0.632

For each target, based on the 58/21 mutant and the unmutated sgRNA sequences, we evaluate the Pearson correlation between the experimentally determined SpCas9 cleavage efficiency^14, 19, 23, 24^ and the computationally predicted (a) stability/population of the three functional structure candidates or (b) the CRISPR cleavage scores from other tools. In the table, Δ*G* ^*helix*^ is the free energy of the fully-zipped hybrid helix. *P*_*full*–*zip*_, *P*_*bound*_, and *P*_*active*_ are the fractional populations of the fully-zipped (candidate 1), all the bound structures (candidate 2), and the active structures (candidate 3), respectively. The strong and consistent correlations for *P*_*active*_ indicates that the proposed active structures (candidate 3) are the functional structures for Cas9 cleavage after PAM recognition.

**Figure 2:**
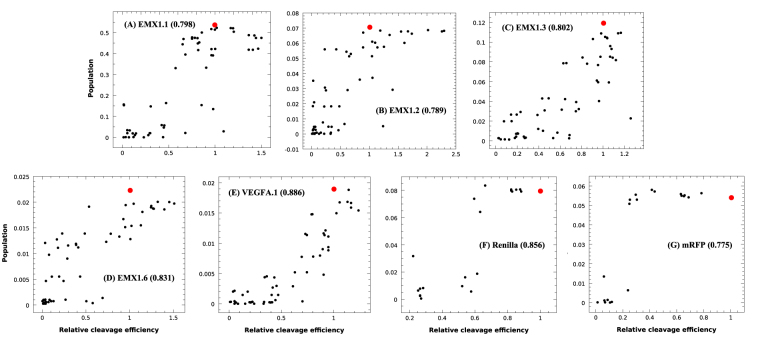
Pearson correlation between the relative cleavage efficiency and the fractional population of functional (active) structures, namely, R-loop structures that contain base pair (1, −1) and at least 7 base pairs in the hybrid helix, for (**A**) EMX1.1, (**B**) EMX1.2, (**C**) EMX1.3, (**D**) EMX1.6, (**E**) VEGFA.1, (**F**) Renilla, and (**G**) mRFP. The numbers in the brackets are the Pearson correlation coefficients. The red dots denote the data for the unmutated sgRNA sequences.

In contrast, as shown in Table 1 and Fig. 2, there exists a strong and consistent correlation for candidate 3 above with *S*_*min*_ = 7. The Pearson correlation ranges from 0.775 to 0.886 with the average of 0.82 for the test cases, indicating that the proposed active structures are likely the functional structures for Cas9 cleavage.

The conclusion about the functional structures, derived from the correlation between structure (from theory) and activity (from experiment), is a result of the Cas9/DNA/sgRNA interactions in the sgRNA target recognition process. Previous studies^12, 13^ suggested that after Cas9 binds to the PAM, the guide RNA invades into the PAM-adjacent protospacer DNA duplex, causing the formation of an R-loop motif immediately upstream of the PAM. During this strand invasion process, the guide RNA must displace the complementary strand of the protospacer. The competition between the invasion and the re-annealing of the DNA duplex results in a dynamic (“breathing”) R-loop structure. PAM recognition and the resultant juxtaposition of the DNA duplex and sgRNA induce the base pairing of (1, −1) immediately upstream of the PAM sequence. To further promote the formation of the double-stranded breaks (DSBs) and cleavage for gene editing, a long hybrid helix (>7 base pairs) is required to stabilize the active structure. For the system shown in Fig. 1, there exist 269 such functional structures out of a total of 17,974 bound structures.

### R-loop energy landscape and CRISPR activity

**Figure 3:**
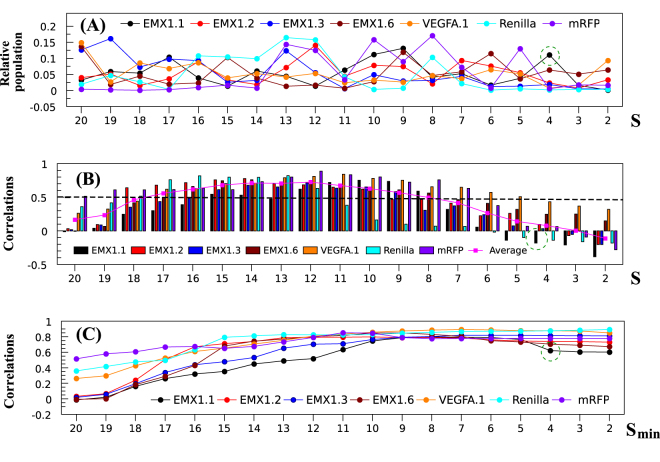
(**A**) Relative populations of the different sgRNA-DNA bound structures out of the total population of the bound structures. A sgRNA-DNA bound structure *M* is characterized by the helix length S base pairs measured from the (1, −1) base pair, as shown in Fig. 1B. For each target, we compute the population distribution *P*_*M*_ for the unmutated sgRNA sequence, and the relative population ${P}_{M}/{e}^{-{G}_{bound}/{k}_{B}T}$ (y-axis). (**B**) Correlation *r*(*P*_*M*_, *F*) between the population *P*_*M*_ of each individual bound structure M, which, as in (**A**), is characterized by the helix length *S* (in base pairs), and the CRISPR cleavage efficiency *F* for the given targets^13, 18^. Here, for each target, for a given *S*, the correlation is evaluated based on all the 58 unmutated and mutated (single mismatch) sgRNA sequences. (**C**) Same as (**B**) except that the population for a given *S*_*min*_ is the sum over all the structures with the helix length longer than *S*_*min*_ base pairs. The sudden decrease at *S*_*min*_ = 4 base pairs (highlighted by the green circle) for EMX1.1 is caused by the negative correlation, shown in Fig. 3B.

First, as shown in Fig. 3A, the unmutated sgRNA sequence shows a bumpy folding free energy landscape ${\rm{\Delta }}{G}_{M}^{f}$ as a function of the different bound structures “M”. Here, structure “M” is described by the length *S* of the sgRNA-DNA duplex zipped from the PAM site. Moreover, the different sgRNA-target systems show lacks a consistent *S*-dependence in the shape of the free energy landscape, suggesting a sensitive sequence dependence of the landscape. As a result, we expect that mutations in the sgRNA sequence may cause notable changes in CRISPR activity.

Second, to investigate the relationship between CRISPR efficiency and the different sgRNA-DNA structures, we evaluate the correlation coefficient *r*(*P*_*active*_, *F*) between the (theoretically predicted) fractional population *P*_*M*_ of a given sgRNA-DNA bound structure M (described by the helix length *S*) and the experimentally measured CRISPR cleavage efficiency. For each target, the correlation for a given M (helix length *S*) is an average over all the 58 sgRNA sequences, including one unmutated and 19 × 3 = 57 single-mutant sequences. The result leads to the following three conclusions (Fig. 3B). (1) Short helices with length S < 7 base pairs yield low or negative correlations. Such structures may be kinetically important as the initial steps in helix formation, however, the short-helix structures are not sufficiently stable and are unlikely the functional structures. (2) Long-helix structures alone may not always give high correlations. For example, structures of *S* > 17 base pairs show weak (<0.5) correlations. (3) The strongest correlation (>0.5) occurs to structures with helix length *S* from 8 to 17 base pairs. The above results suggest that an ensemble of R-loop with RNA-DNA hybrid helix length *S* > 7 base pairs may correspond to the functional structures.

To further confirm the above identified functional structure, we compute the correlation between the activity and the population of the different structure groups, namely, structures of helix length from *S*_*min*_ to 20 base pairs. As shown in Fig. 3C, the correlation reaches a plateau at *S*_*min*_ between 10 to 15 base pairs for the tested systems. The result indicate that for certain sgRNA and target sequences, a protospacer of length 15–20 bps might be sufficient to provide high cleavage efficiency^33, 34^.

Experiments suggest that the 20-nt long protospacer can be divided into two regions, the seed (PAM-proximal) region within 10 base pairs from the PAM and the non-seed (PAM-distal) region with 10 base pairs away from the PAM. Cas9 tolerates single-base mismatches in the non-seed region to a greater extent than in the seed region^19^. The position-dependent mismatch tolerance can also be explained by the statistical mechanical analysis above. Figure 3C shows the strong correlation between Cas9 cleavage and structures of helix length from 8 to 20 base pairs. When a single mismatch is introduced in the seed region, nearly all the functional structures contain the mismatch and hence the total cumulative population of these structures is greatly affected, resulting in a large change in the cleavage efficiency. However, if a single mismatch is introduced in the non-seed region, only a fraction of the functional structures contains the mismatch, thus, the impact to the total population of the functional structures is small. Therefore, mismatches in the non-seed region would have less impact on the CRISPR activity than mismatches in the seed region.

### Prediction of off-target sites

To compare our method with the other existing methods, such as CROP-it^17^, CCTop^18^, Zhang’s model^19^, and CFD^21^, we compute the correlation between the theoretical cleavage efficiency metric, such as the total population *P*_active_ of the functional structures in our current new method and the experimentally measured CRISPR efficiency. The test results shown in Table 1 for the aforementioned seven gene target systems, each with 58/21 sgRNA sequences, indicate that the different methods generally give reasonably consistent correlations except CCTop, which has negative correlations due to its specific scoring algorithm.

The unique feature of our approach is to account for the effect from not only the single “native” state with the fully zipped 20-bp sgRNA-DNA helix, but also the full spectrum of the functional states on the energy landscape, including the nonnative (bound and unbound) and partially folded sgRNA-DNA structures. The question, however, is whether these existing algorithms and our current new method can correctly assess the genome-wide off-target effects, which often involve multiple mismatches. Here, we use 24 cases obtained from four published data sets^11, 19, 29, 30^ to train the parameters and to test the reliability of the algorithms for off-target assessment. We also compare our new method with other existing methods^17–19, 21^.

Table 2 shows the test results for the above mentioned 24 cases. The tested sgRNAs have different numbers of off-targets, ranging from 9 to 5,957 genome-wide. For each sgRNA sequence, we calculate *P*_active_, the total fractional population of the functional structures, for each scanned target. Sum over all the on- and off-targets gives the Pearson correlation coefficient for the given sgRNA sequence. We find that CROP-it^17^ and CCTop^18^ give similar performances, with most of the off-targets showing weak/no correlations. The algorithm by Hsu *et al*.^19^ (Zhang’s model) gives improved correlations, with 10 out of 24 cases showing strong (>0.5) correlations. Without the mismatch sequence dependence, CROP-it, CCTop, and the Zhang lab’s scoring metrics may not correctly capture the effects of multiple-mismatches. For the CFD^21^ scoring method, 17 out of 24 cases show strong (>0.5) correlations. Only 7 cases, with large numbers of off-targets, have the correlations below 0.5. The improvement of the performance may be attributed to the usage of the mismatch sequence- and position-dependent scoring functions. However, all four existing tools consider only mismatches in the full-length hybrid helix and cannot treat sgRNA/DNA sequences involving bulges and other loops in the sgRNA-DNA duplex^22^.

**Table 2:** The Pearson correlations of six off-target scoring methods with the experimentally measured genome-wide off-target activities.

Target	# of off-targets	CROP-it	CCTop	Zhang’s model	CFD	SMA_none_	SMA_para_
From Hsu *et al*.^19^							
EMX1.1	9	0.799	−0.402	0.974	0.979	0.998	0.981
EMX1.3	33	0.227	−0.18	0.221	0.723	0.552	0.889
From Kuscu *et al*.^29^							
sgRNA1	50	0.327	−0.288	0.698	0.788	0.789	0.851
sgRNA2	17	0.466	−0.234	0.532	0.911	0.632	0.744
sgRNA3	41	0.195	−0.194	0.462	0.908	0.891	0.976
sgRNA4	484	0.077	−0.046	0.072	0.135	0.094	0.501
sgRNA5	52	0.343	−0.134	0.088	0.749	0.859	0.872
sgRNA6	1282	0.064	−0.026	0.228	0.251	0.233	0.301
sgRNA7	285	0.14	−0.039	0.614	0.673	0.216	0.639
sgRNA8	43	0.543	−0.171	0.641	0.812	0.548	0.501
sgRNA9	121	0.331	−0.062	0.825	0.804	0.861	0.812
sgRNA10	202	0.124	−0.169	0.773	0.76	0.777	0.579
sgRNA11	16	0.474	−0.148	0.643	0.649	0.642	0.628
sgRNA12	14	0.818	−0.009	0.82	0.832	0.818	0.818
From Tsai *et al*.^30^							
VEGFA(1)	22	0.448	−0.556	0.068	0.819	0.469	0.613
VEGFA(2)	151	0.298	−0.289	0.499	0.434	0.649	0.501
VEGFA(3)	60	0.332	−0.196	0.189	0.542	0.514	0.661
EMX1	16	0.352	−0.29	0.595	0.723	0.694	0.689
FANCF	9	0.375	−0.589	0.366	0.927	0.813	0.776
HEK293(4)	134	0.259	−0.131	0.404	0.379	0.177	0.501
From Wu *et al*.^11^							
Nanog-sg2	26	0.294	−0.335	0.428	0.808	0.311	0.502
Nanog-sg3	5957	0.065	−0.123	0.067	0.078	0.082	0.209
Phc1-sg1	2948	0.179	−0.263	0.163	0.207	0.176	0.342
Phc1-sg2	663	0.199	−0.168	0.245	0.271	0.186	0.394
Success rate*		3/24	2/24	10/24	17/24	14/24	20/24

Here, SMA_none_, and SMA_para_ are the predicted scores (populations) from our statistical mechanical analysis (SMA)-based model without and with additional parameters, respectively. The experimental data is from the published papers.

*Percentage of cases that show high (>0.5) correlation.

The genome-wide test results (Table 2) support the conclusion that our new statistical mechanics-based algorithm provides improved off-target assessment. To further confirm the physical mechanism, namely, the relationship between the stability of the identified functional structures and the CRISPR activity, we test the algorithm at the different levels. As the lowest approximation, we first consider only the contributions from the canonical base pairs/stacks in our statistical mechanical analysis (SMA) (see SMAnone in Table 2). As shown in Table 2, 14 out of 24 cases show strong (>0.5) correlations. Without using any fitting parameters, the SMAnone scoring metric outperforms Zhang lab’s model and reaches similar performance to CFD, which employs 240 fitted parameters. The results suggest that our algorithm may have captured the structure-function relationship for the Cas9/DNA/sgRNA system. For targets with large numbers of off-target sites, such as Nanog-sg3, which has 5,957 off-target sites, none of the scoring metrics can provide satisfactory correlation. To further increase the prediction accuracy, we introduce 261 energy parameters for the different mismatched base pairs as well as the position dependence of the mismatches. The parameters are estimated based on the optimization of the aforementioned correlation coefficient for the data listed in Table 2. The model with additional energy parameters (SMApara) gives improved predictions for the off-targets, where 20 out of 24 cases show strong correlations.

**Figure 4:**
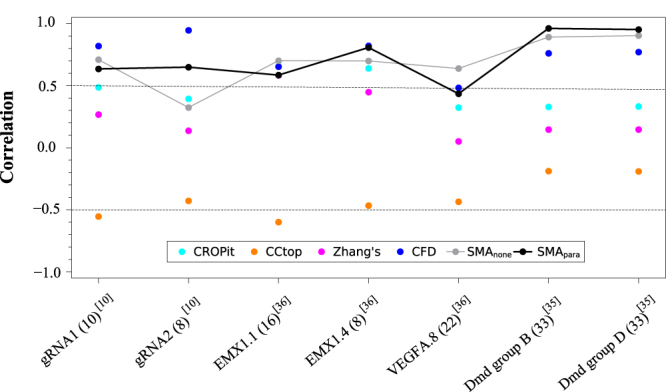
Tests of six off-target scoring methods using the experimentally measured genome-wide off-target activities. The numbers shown in the brackets are the number of the off-targets. The experimental data are from the corresponding published references. SMA_none_ and SMA_para_ are our models with and without parameters, respectively.

We also use seven cases obtained from three published data sets^10, 35, 36^ to benchmark test the predictive power of our model. We note that these seven test cases are not included in the training set (24 cases in Table 2). As shown in Fig. 4, our statistical mechanical analysis-based models (without any parameter, SMAnone, and with additional energy parameters, SMApara) can have comparable or better performances than other non-physical algorithms, indicating the current models may possibly capture important aspects of the physical mechanism for CRISPR/Cas9 gene editing. Furthermore, with increasing amount of experimental data, our physical mechanism-based model, by incorporating more reliable parameters, may offer continuously improving predictions for off-target assessment and optimal sgRNA design.

To implement the new algorithm described above, we have developed a user-friendly computational tool (VfoldCAS) to predict/rank off-target loci to facilitate the sgRNA design. The tool can be accessed at http://rna.physics.missouri.edu/vfoldCAS. With the increasing amount of available experimental CRISPR data, the model is expected to provide off-target site predictions and sgRNA design with increasing accuracies.

## Conclusions

Based on statistical mechanical principles and CRISPR gene editing efficiency data for different targets and sgRNAs, we explore the structure-based physical mechanism of the two-stage CRISPR gene editing process. The first stage is PAM recognition. This stage is determined by the PAM sequence and PAM/Cas9 interactions and chromatin accessibility. The second stage is the formation of the R-loop, namely, the target DNA/sgRNA bound structure. Different R-loop structures have different contributions to the overall cleavage efficiency. Through extensive theory-experimental comparisons we reveal a strong correlation between the population (stability) of the functional bound structures and the Cas9 cleavage efficiency. Such a correlation suggests that the folding stability of the functional structures plays an important role in the DNA targeting specificity of the CRISPR/Cas9 system. Specifically, we find that the major contribution comes from the bound structures which contain DNA-sgRNA helices of length 8–17 base pairs zipped from the PAM terminal. Our finding suggests that a full length (20 base pairs) RNA-DNA hybrid helix may not be mandatory for sgRNA-target recognition and Cas9 cleavage, and shorter protospacers can also ensure high targeting efficiency. Furthermore, the result supports the conclusion that Cas9 can tolerate mismatches in the PAM-distal (non-seed) region, although the perfect base-pairing in the PAM-proximal (seed) region is preferred.

From the kinetics point of view, the sgRNA-DNA hybrid helix can be quickly zipped up from the initially formed base pairs at the PAM binding site such as the (1, −1) base pair in Fig. 1B. However, from our statistical mechanical analysis, we find no correlation between the formation of the initial base pairs (a short hybrid helix) in the PAM-proximal region and CRISPR activity. The lack of correlation may stem from two possible reasons. First, as indicated in previous single-molecule DNA supercoiling experiments^12^ and AFM imaging with kinetic Monte Carlo simulations^13^, sgRNA-DNA helix folding kinetics involves a “breathing” process, where short-helix R-loop structures are transiently folded and unfolded. Second, Cas9 cleavage may require a sufficiently long hybrid helix for the double-stranded breaks. Therefore, as shown in Fig. 3B, the short-helix R-loop structures are unlikely functional and thus show low or negative correlations with CRISPR activity. Once the hybrid helix exceeds a threshold length, the sgRNA-DNA helix would proceed to zip up to perform the cleavage function.

Our current model, which uses the total population of the ensemble of functional R-loop structures zipped unidirectionally from the PAM-adjacent base pairs implicitly taken the kinetics pathway effect into account. In addition, as shown in Fig. 2, the unmutated sgRNA sequences have a high cleavage efficiency as expected, while some mutant sgRNAs even have better efficiency than the unmutated ones, suggesting possible additional effects, such as the 3D structure and the sequence-specific effects, beyond sgRNA-DNA base pairing. Further improvement of the model should consider the Cas9/PAM related structural features.

The identified physical mechanism leads to a new method for predicting CRISPR off-target sites and optimal design of sgRNAs for a given target. Unlike previous methods, which often involve ad hoc data fitting, this current new method is based on a rigorous physical mechanism. Thus, it can provide more accurate predictions. Indeed, tests with genome-wide data indicate that our new model gives more accurate predictions on off-targets than other existing scoring metrics. This new algorithm may offer an accurate method for optimal sgRNA design that can maximize activity and minimize off-target effects.

The new method reported here also has the unique ability to treat general R-loop structures. For instance, unlike previous methods, the current method considers contributions from bulge-looped structure. Further development of the method includes the consideration of a more complete ensemble of R-loop structures. Moreover, correct energy and entropy parameters are essential for the further development of the physical model. As more and more data become available, we expect a continuous increase in the number of available energy and entropy parameters. Chromatin accessibility can influence sgRNA-target binding. However, none of the currently available off-target site prediction tools can consider the spatial accessibility of the site in the 3D genome structure. Future model development should also consider additional potentially important factors such as the chromatin accessibility in PAM recognition and other possible effects such as the kinetic effects in R-loop formation and subsequent DNA/RNA hybridization, and the torque (twisting force induced by the DNA supercoiling)-regulated R-loop formation and disruption of the Cas9 cleavage efficiency.

## Electronic supplementary material

Supplementary PDF File
