# Supplementary material for: CRISPR-Cas9 cleavage efficiency correlates strongly with target-sgRNA folding stability: from physical mechanism to off-target assessment
Source: Sci Rep. 2017 Mar 10;7:143. doi: 10.1038/s41598-017-00180-1 (PMC5427927; doi:10.1038/s41598-017-00180-1)
Supplement: Supplementary file 1 — Supplementary PDF File [file 41598_2017_180_MOESM1_ESM.pdf]

Supplementary Information:

CRISPR-Cas9 cleavage efficiency correlates strongly with target-sgRNA folding stability: from physical mechanism to off-target assessment

Xiaojun Xu[1]**, Dongsheng Duan**[2]**, Shi-Jie Chen**[1]

1. Department of Physics, Department of Biochemistry, and Informatics Institute, University of Missouri, Columbia, MO, USA
2. Department of Molecular Microbiology and Immunology, Department of Neurology, School of Medicine; Department of Biomedical Sciences, College of Veterinary Medicine; and Department of Bioengineering, University of Missouri, Columbia, MO, USA

[1] Schematic of the experimental design from Zhang's lab

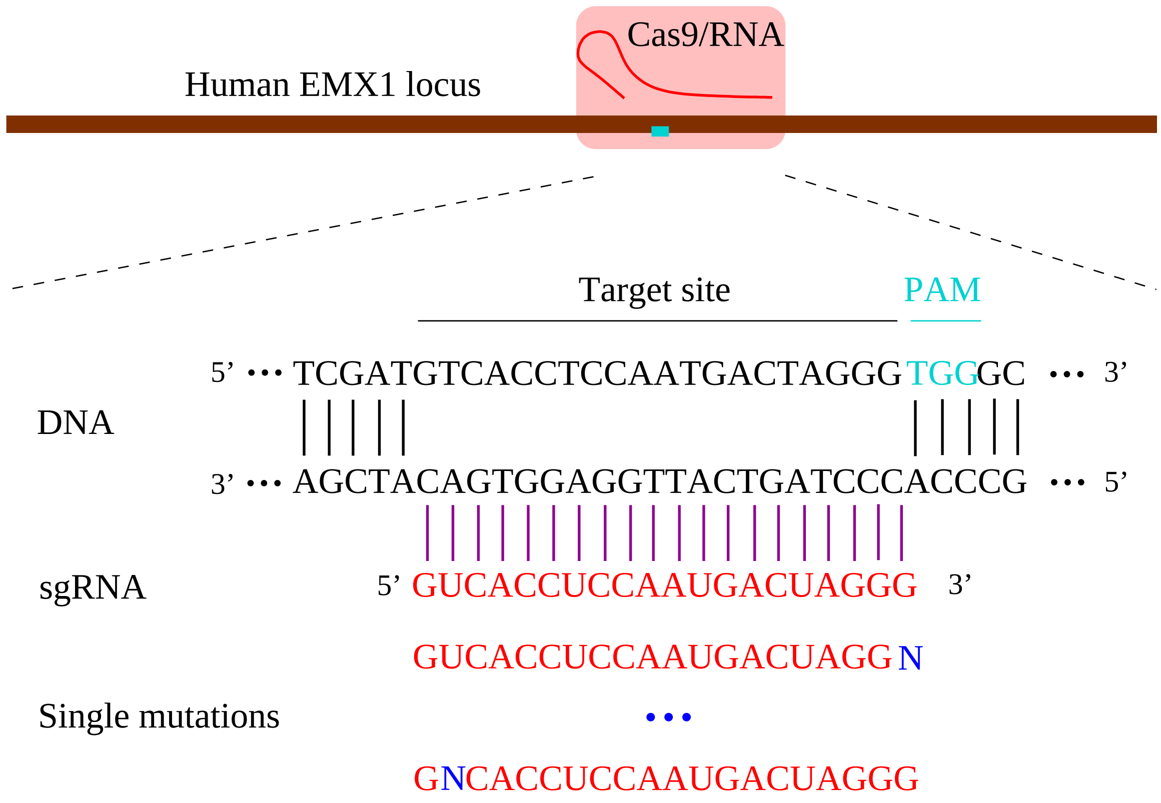

Figure S1: Schematic of the experimental design from Zhang's lab [1, 2]. sgRNAs carrying all possible single base pair mismatches throughout the guide sequence were tested for each target sites.

[2] Structural ensemble

Table S1: Comparison of the Pearson correlations between the calculated populations and the experimental measured single-nucleotide specificity of SpCas9. As shown in Fig.1B, there are two different ways to (1) all binding modes for the hybrid helix zipped from the (1,-1) base pair, which leads to 291 bound structures. enumerate bound (DNA/RNA complexes) structures: (2) all possible binding modes for the hybrid helix, which leads to 17974 bound structures. There is only one unbound structure (Fig.1) for both cases. The functional (active) structures are the bound structures containing the (1,-1) base pair. and having the length of $>$ 7 base pairs.

Target	$P^{(1)}_{\mathbf{active}}$	$P^{(1)}_{\mathrm{bound}}$	$\mathbf{P}^{(2)}_{\mathbf{active}}$	$P^{(2)}_{\mathrm{bound}}$
	269/292	291/292	269/17975	17974/17975
EMX1.1	0.801	0.569	**0.798**	0.782
EMX1.2	0.798	0.769	**0.789**	0.532
EMX1.3	0.786	0.807	**0.802**	0.387
EMX1.6	0.809	0.718	**0.831**	-0.046
VEGFA.1	0.868	0.836	**0.886**	-0.426
Renilla	0.691	0.714	**0.856**	0.201
mRFP	0.773	0.778	**0.775**	0.624
Average	0.789	0.742	**0.820**	0.293

[3] R-loop contributions for the case of 292 total structures

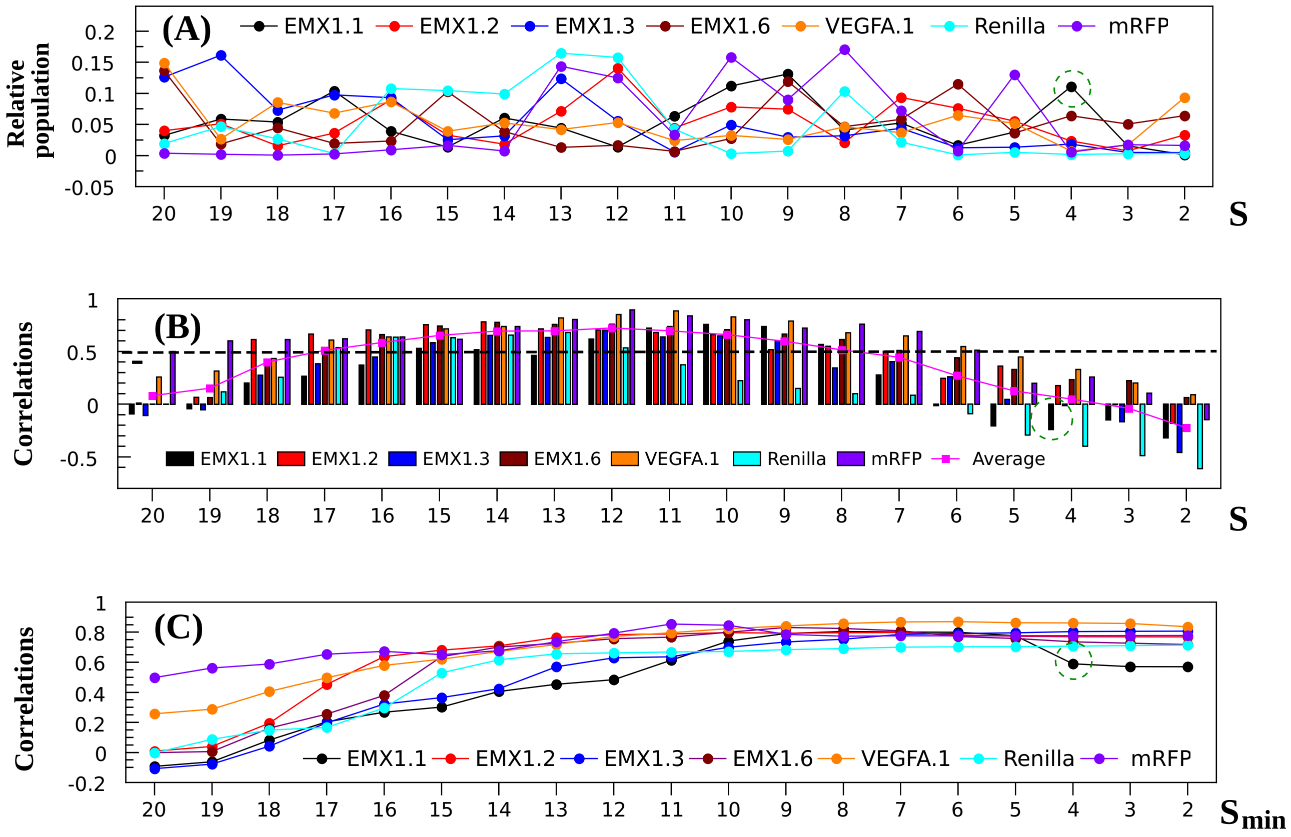

Figure S2: (A) Relative populations of the different sgRNA-DNA bound structures (characterized by the helix length S measured from the (1,-1) base pair between the sgRNA and the DNA, shown in Fig. 1B, with respect to the overall population of all the sgRNA-DNA bound structures that contains the hybrid base pair (1, -1). For each target, we compute the population distribution for the unmutated sgRNA sequence, $Z_S / \sum_S Z_S$ with Z_S the partition function of all bound structures of S. (B) Correlations between the population of each individual bound structure, which, as in (A), is characterized by the helix length S, $P_S = Z_S/Z_{tot}$ and the CRISPR cleavage efficiency for the given targets [1, 2]. Here, for each target, for a given S, the correlation is evaluated based on all the 58/21 unmutated and mutated (single mismatch) sgRNA sequences. (C) Same as (B) except the population for a given S_{min} is the sum over all the structures with helix length longer than S_{min} base pairs. Compared with the case of all bound structures (17975), shown in Fig.3, there are only small changes due to the choice of the different bound structures.

[4] Off-target scoring without additional parameters

Table S2: Comparison of the Pearson correlations between the two choices of the bound structures for the genome-wide off-target scoring. Without additional parameters, the two cases have similar performances.

Target	# of off-targets	SMA^{292}_{none}	SMA^{17975}_{none}
From Wu et. al. [3]			
Nanog-sg2	26	0.31	0.245
Nanog-sg3	5957	0.083	0.079
Phc1-sg1	2948	0.176	0.177
Phc1-sg2	663	0.175	0.17
From Hsu et. al. [1]			
EMX1.1	9	0.998	0.998
EMX1.3	33	0.552	0.455
From Kuscu et. al. [4]			
sgRNA1	50	0.79	0.469
sgRNA2	17	0.632	0.283
sgRNA3	41	0.879	0.713
sgRNA4	484	0.082	0.075
sgRNA5	52	0.859	0.783
sgRNA6	1282	0.233	0.227
sgRNA7	285	0.225	0.213
sgRNA8	43	0.548	0.323
sgRNA9	121	0.86	0.83
sgRNA10	202	0.775	0.759
sgRNA11	16	0.642	0.641
sgRNA12	14	0.818	0.819
From Tsai et. al. [5]			
VEGFA(1)	22	0.48	0.715
VEGFA(2)	151	0.65	0.539
VEGFA(3)	60	0.518	0.503
EMX1	16	0.694	0.596
FANCF	9	0.82	0.667
HEK293(4)	134	0.177	0.167

[5] Definition of parameters

As listed below, we define three types of parameters for the CRISPR/Cas9 scoring. There are 261 parameters (240 mismatched base stacking energy terms, 1 bulge-loop energy term, and 20 mismatch efficiency reductions) in total. To train the parameters using the random search method and the experimental determined genome-wide off-target data, we restrict the values of the parameters in the ranges of: (-2, 2) kcal/mol for the mismatched base stack, (0, 4) kcal/mol for the bulge loop, and (0, 1) for the mismatch-related efficiency reduction.

1. Mismatched base stacks, as shown in Fig.S3 (A). There are 240 different types of mismatched base stacks, corresponding to the free energies of the formation of the sequence-dependent but position-independent base stacks in the DNA/RNA hybrid helix.

2. One nucleotide-bulge, as shown in Fig.S3 (B). This is a position- and sequence-independent energy term, corresponding to the entropic free energy for the formation of the one-nucleotide bulge loop.

3. Mismatched base pair induced efficiency reduction, as shown in Fig.S3 (C). This is a position-dependent but sequence-independent efficiency factor, related to the extent of Cas9 tolerance to mismatches.

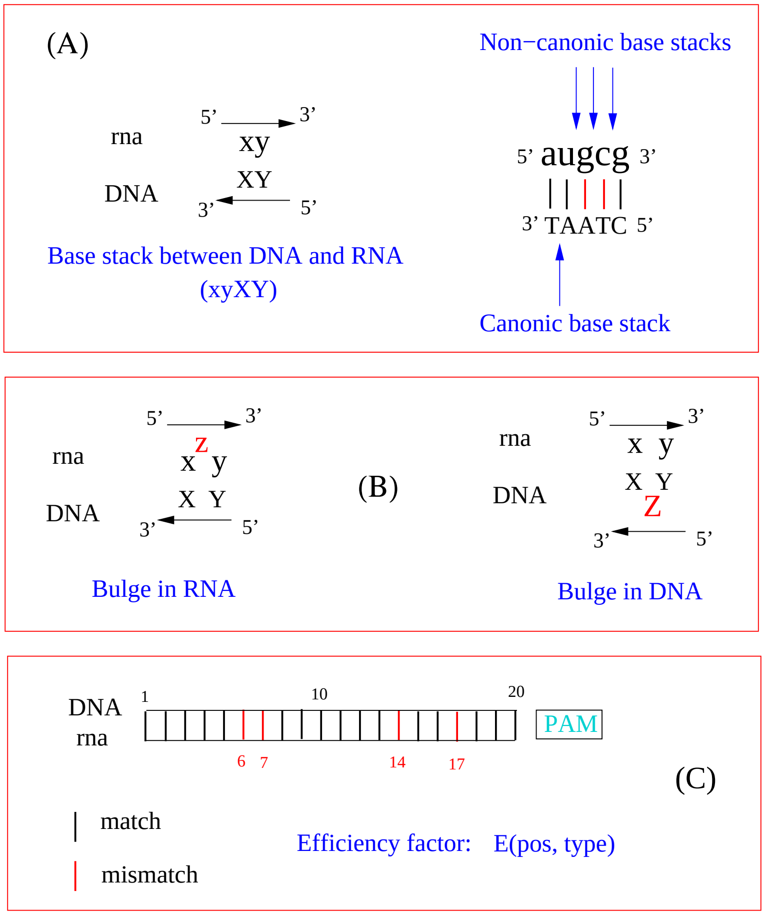

Figure S3: Definition of parameters. (A) Base stacks between DNA and RNA. We use xyXY to denote each stack, with lower letters for RNA and upper letters for DNA. In total, there are 240 non-canonical base stacks. (B) Bulge loop in either RNA or DNA strand. (C) Position-dependent mismatched base pair-induced efficiency factor. E(pos,match) = 1.0 for matched base pairs, regarding the positions. As an example, there are four mismathes at positions (6, 7, 14, and 17).

Table S3: Values of the parameters. The units of the mismatched stacks and the bulge loop are in kcal/mol. The efficiency factor is unitless.

Type	Value	Type	Value	Type	Value	Type	Value	Type	Value	Type	Value	Type	Value
aaAA	-0.8	aaAT	-0.9	aaAG	-2	aaAC	-1.9	aaTA	-0.7	aaTG	-2	aaTC	-1
aaGA	0.6	aaGT	-0.8	aaGG	-1.9	aaGC	-1.3	aaCA	-1	aaCT	-1.1	aaCG	-2
aaCC	-2	auAA	-0.3	auAG	-1.9	auAC	-1.6	auTA	-1.4	auTT	-0.1	auTG	-1.9
auTC	-1.2	auGA	-0.8	auGT	0.1	auGG	-2	auGC	-2	auCA	-2	auCT	-1.7
auCG	-2	auCC	-2	agAA	0.7	agAT	-0.8	agAG	2	agAC	-1.9	agTA	-1.7
agTT	-0.8	agTG	-2	agTC	-2	agGA	-1.3	agGT	-1.9	agGG	-1.5	agGC	-0.9
agCA	-0.4	agCG	2	agCC	-1.2	acAA	-1.7	acAT	0	acAG	-0.2	acAC	-1.5
acTA	-2	acTT	1.8	acTG	-2	acTC	-0.8	acGA	-2	acGG	-1.7	acGC	-1.9
acCA	-2	acCT	-2	acCG	-1.9	acCC	-2	uaAA	-1.2	uaAT	-1.9	uaAG	-1.2
uaAC	-1.4	uaTT	-0.9	uaTG	2	uaTC	0	uaGA	-1.3	uaGT	-1	uaGG	-2
uaGC	-1.3	uaCA	-1.3	uaCT	2	uaCG	-1.8	uaCC	-1.7	uuAT	2	uuAG	-1.4
uuAC	2	uuTA	2	uuTT	-2	uuTG	-2	uuTC	2	uuGA	1.5	uuGT	2
uuGG	-2	uuGC	2	uuCA	2	uuCT	-2	uuCG	-2	uuCC	2	ugAA	-1.4
ugAT	-1.7	ugAG	-2	ugAC	-0.2	ugTA	-0.6	ugTT	0.3	ugTG	-2	ugTC	1.9
ugGA	-1.3	ugGT	-0.1	ugGG	-2	ugGC	-1.8	ugCT	-2	ugCG	-2	ugCC	-2
ucAA	-2	ucAT	-1.3	ucAG	-0.6	ucAC	-2	ucTA	2	ucTT	0.4	ucTG	-2
ucTC	-0.5	ucGT	2	ucGG	-2	ucGC	2	ucCA	2	ucCT	0.1	ucCG	-1
ucCC	-0.6	gaAA	-0.3	gaAT	2	gaAG	-0.7	gaAC	0.4	gaTA	-0.4	gaTT	-1.2
gaTG	-2	gaGA	-0.9	gaGT	-1.1	gaGG	2	gaGC	-1.9	gaCA	-1.9	gaCT	-1.6
gaCG	-1.7	gaCC	-2	guAA	-1.4	guAT	-0.5	guAG	-2	guTA	-1.1	guTT	-0.8
guTG	-1.4	guTC	-0.8	guGA	-2	guGT	-0.7	guGG	-1.1	guGC	1.8	guCA	2
guCT	-1.8	guCG	-0.6	guCC	-0.7	ggAA	-0.2	ggAT	-2	ggAG	-1	ggAC	2
ggTA	-2	ggTT	1	ggTG	-2	ggTC	-0.8	ggGA	-2	ggGT	-2	ggGG	-2
ggGC	-0.9	ggCA	-2	ggCT	-1.9	ggCG	1.9	gcAA	-1.5	gcAT	-2	gcAG	-2
gcAC	-0.8	gcTA	2	gcTT	-2	gcTG	-2	gcTC	-2	gcGA	-1.9	gcGT	-2
gcGG	-2	gcCA	-2	gcCT	-1.6	gcCG	-2	gcCC	-2	caAA	-0.6	caAT	-1.5
caAG	-1	caAC	-1.9	caTA	1.9	caTT	2	caTC	-0.9	caGA	-1.1	caGT	-1.9
caGG	0.1	caGC	-2	caCA	-1	caCT	-1.8	caCG	-2	caCC	-0.8	cuAA	-1.7
cuAT	-1.2	cuAC	-1.2	cuTA	-1.9	cuTT	-0.1	cuTG	-2	cuTC	-1.3	cuGA	-2
cuGT	-1.8	cuGG	-1.9	cuGC	-2	cuCA	-2	cuCT	0.3	cuCG	0.5	cuCC	-2
cgAA	2	cgAT	-2	cgAG	-2	cgAC	2	cgTA	-2	cgTT	-1	cgTG	-1.5
cgTC	-1.2	cgGA	-2	cgGT	-1.7	cgGG	-2	cgGC	-2	cgCA	-1.9	cgCT	-1.7
cgCC	-2	ccAA	-2	ccAT	2	ccAG	-1.1	ccAC	-2	ccTA	-2	ccTT	-2
ccTG	-1.3	ccTC	2	ccGA	2	ccGT	2	ccGC	-1.5	ccCA	-2	ccCT	-2
ccCG	2	ccCC	-2	Bloop	0.9	E(20)	1.0	E(19)	1.0	E(18)	1.0	E(17)	0.7
E(16)	0.2	E(15)	0.05	E(14)	0.4	E(13)	0.1	E(12)	0.2	E(11)	0.00001	E(10)	0.2
E(9)	0.04	E(8)	1.0	E(7)	0.7	E(6)	0.5	E(5)	0.3	E(4)	0.00001	E(3)	0.4
E(2)	0.00001	E(1)	0.00001										

References

[1] Hsu PD, Scott DA, Weinstein JA, Ran FA, Konermann S, Agarwala V, Li Y, Fine EJ, Wu X, Shalem O, Cradick TJ, Marraffini LA, Bao G, Zhang F. (2013). DNA targeting specificity of RNA-guided Cas9 nucleases. *Nat Biotechnol*, **31**(9), 827-832.

[2] Slaymaker IM, Gao L, Zetsche B, Scott DA, Yan WX, Zhang F. (2016). Rationally engineered Cas9 nucleases with improved specificity. *Science*, **351**(6268), 84-88.

[3] Wu X, Scott DA, Kriz AJ, Chiu AC, Hsu PD, Dadon DB, Cheng AW, Trevino AE, Konermann S, Chen S, Jaenisch R, Zhang F, Sharp PA. (2014). Genome-wide bound of the CRISPR endonuclease Cas9 in mammalian cells. *Nat Biotechnol*, **32(7)**, 670-676.

[4] Kuscu C, Arslan S, Singh R, Thorpe J, Adli M. (2014). Genome-wide analysis reveals characteristics of off-target sites bound by the Cas9 endonuclease. *Nat Biotechnol*, **32**(7), 677-683.

[5] Tsai SQ, Zheng Z, Nguyen NT, Liebers M, Topkar VV, Thapar V, Wyvekens N, Khayter C, Iafrate AJ, Le LP, Aryee MJ, Joung JK. (2015). GUIDE-seq enables genome-wide profiling of off-target cleavage by CRISPR-Cas nucleases. *Nat Biotechnol*, **33**(2), 187-197.
